# Association of *PON1* Q192R/L55M Polymorphisms and Paraoxonase-1 levels with cardiometabolic risk in patients with type-2 diabetes mellitus

**DOI:** 10.12669/pjms.42.7.15469

**Published:** 2026-07

**Authors:** Ahmed M Ahmed, Hakeemah H Al-Nakhle, Kholoud Ashour, Hashim M Aljohani, Zakaria Eltahir

**Affiliations:** 1 Ahmed M Ahmed, Department of Clinical Laboratory Sciences, College of Applied Medical Sciences, Taibah University - Madinah, 41311, Kingdom of Saudi Arabia; 2 Hakeemah H Al-Nakhle, Department of Clinical Laboratory Sciences, College of Applied Medical Sciences, Taibah University - Madinah, 41311, Kingdom of Saudi Arabia; 3 Kholoud Ashour, Department of Clinical Laboratory Sciences, College of Applied Medical Sciences, Taibah University - Madinah, 41311, Kingdom of Saudi Arabia; 4 Hashim M Aljohani, Department of Clinical Laboratory Sciences, College of Applied Medical Sciences, Taibah University - Madinah, 41311, Kingdom of Saudi Arabia; 5 Zakaria Eltahir, Department of Clinical Laboratory Sciences, College of Applied Medical Sciences, Taibah University - Madinah, 41311, Kingdom of Saudi Arabia

**Keywords:** Cardiovascular disease, L55M, Paraoxonase-1, *PON1*, Q192R, T2DM

## Abstract

**Objectives:**

To investigate the distribution of *PON1* Q192R and L55M polymorphisms and serum PON1 concentration in Saudi patients with T2DM and to evaluate their associations with biochemical parameters and diabetes-related complications.

**Methodology:**

In this case–control study, 220 patients with T2DM and 120 healthy controls were enrolled from several hospitals in Madinah, KSA, from May 1, 2025 to December 15, 2025. Clinical and biochemical assessments included fasting plasma glucose (FPG), HbA1c, lipid profile, apolipoprotein A1 (ApoA1), and apolipoprotein B (ApoB). *PON1* Q192R and L55M genotypes were determined using PCR–RFLP, and serum PON1 concentration was measured by ELISA. Associations between genotypes, PON1 levels, metabolic indices, and diabetic complications were analyzed.

**Results:**

Serum PON1 levels were significantly lower in patients with T2DM compared with controls (p < 0.001). The genotype and allele frequencies of *PON1* Q192R and L55M polymorphisms did not differ significantly between groups (*p*=0.18); Q192R allele: (*p*=0.21); L55M genotype: (*p*=0.58); L55M allele: (*p*=0.43). While neither polymorphism was associated with T2DM susceptibility, the Q192R variant was significantly associated with cardiovascular disease among patients with T2DM (*p* = 0.04). Both polymorphisms were significantly associated with serum PON1 concentration and glycemic indices (FPG and HbA1c; *p* < 0.05), whereas no significant associations were observed with BMI or lipid parameters.

**Conclusion:**

In Saudi patients with T2DM, PON1 levels are notably lower, indicating reduced antioxidant defense. Although certain genetic polymorphisms of PON1 do not affect diabetes susceptibility, the Q192R variant may increase cardiovascular risks in T2DM, suggesting its role as a biomarker for cardiometabolic risk rather than disease development.

## INTRODUCTION

Type-2 diabetes mellitus (T2DM) is a significant global public health issue, marked by chronic high blood sugar due to insulin resistance and impaired insulin secretion. This condition affects glucose and lipid metabolism, resulting in multi-organ damage and decreased quality of life.^1^,^2^ Consistent with these observations, the Kingdom of Saudi Arabia (KSA) ranks seventh globally and second in the Middle East in terms of diabetes prevalence, according to the World Health Organization (WHO).^3^ Alarmingly, the prevalence of T2DM in KSA has increased from 16.4% during 2000–2020 to 28% in 2023,^4^ emphasizing the need to identify genetic and biochemical determinants that may contribute to disease susceptibility and cardiovascular risk in this population.^5^

Oxidative stress significantly contributes to the vascular complications of T2DM, where chronic hyperglycemia leads to oxidative damage and cardiovascular risks. Paraoxonase-1 (PON1), an antioxidant enzyme linked to high-density lipoprotein (HDL), helps mitigate this damage by preventing low-density lipoprotein (LDL) oxidation and maintaining endothelial health.^6^

*PON1* is a member of the Paraoxonase gene family, which includes *PON1*, *PON2*, and *PON3*, and is encoded by a gene cluster located on chromosome 7q21.3–q22.1. The PON proteins are approximately 43 kDa glycoproteins comprising about 337 amino acids and exhibit arylesterase and lactonase activities toward a wide range of substrates, including organophosphates and lipid peroxides. In circulation, PON1 is closely associated with apolipoprotein A-I (ApoA-I) within a specific HDL subfraction enriched in ApoA-I and ApoJ, a structural arrangement critical for its stability and enzymatic activity.^7^

Genetic polymorphisms in the *PON1* gene, notably Q192R and L55M, significantly influence enzyme concentration, catalytic efficiency, and antioxidant capacity, contributing to susceptibility to oxidative stress related disorders such as dyslipidemia and cardiovascular disease.^8^

Evidence indicates that individuals with T2DM have reduced PON1 activity compared to none diabetics, possibly worsening oxidative stress and atherogenesis.^9^ Consequently, *PON1* Q192R and L55M polymorphisms have been investigated as genetic modifiers of T2DM risk, lipid metabolism, and cardiovascular complications. However, findings across populations remain inconsistent, and data from Middle Eastern cohorts particularly Saudi populations are limited.

We hypothesized that functional polymorphisms in the *PON1* gene (Q192R and L55M) are associated with altered circulating PON1 levels and contribute to increased metabolic and cardiovascular risk among Saudi patients with T2DM. Specifically, we postulated that risk associated genotypes are linked to reduced PON1 activity, adverse lipid profiles, poorer glycemic control, and a heightened cardiovascular risk phenotype. Therefore, this study investigated the distribution of these polymorphisms and their associations with PON1 concentrations, metabolic parameters, and diabetes-related complications.

## METHODOLOGY

In this case control study, 220 patients with T2DM were diagnosed according to standard criteria (fasting plasma glucose ≥ 126 mg/dL or HbA1c ≥ 6.5%) and recruited from several hospitals in Madinah, Kingdom of Saudi Arabia, from 01/05/2025 to 15/12/2025. The control group comprised 120 healthy subjects with normal glycemic indices. Exclusion criteria included patients with severe illnesses, cancer, liver problems, known autoimmune disorders, and end stage kidney disease requiring dialysis; pregnant women; people with diabetes other than Type-2; and children. Diabetic complications were diagnosed according to previously established criteria.^10^ Nephropathy was diagnosed based on a glomerular filtration rate (GFR) <60 mL/min and a urine albumin to creatinine ratio (UACR) >30 mg/g. Fundus findings (hemorrhages, microaneurysms, and neovascularization) from retinal imaging were used to diagnose retinopathy. Neuropathy was diagnosed according to the American Academy of Neurology, which includes both clinical symptoms and nerve conduction testing. Clinical and laboratory markers were used in accordance with ADA guidelines to diagnose cardiovascular disorders (CVD).

### Biochemical analysis:

PON1 concentration was measured using a commercial ELISA kit (Elabscience, USA) as previously mentioned.^9^ Glucose, lipids, apoA, and apo B levels were measured enzymatically with a Dimension® EXL™ 200 full biochemistry autoanalyzer (Siemens, Erlangen, Germany). HbA1c was measured with a D-10™ Hemoglobin Analyzer manufactured by Bio-Rad (Nyocard, USA).

### DNA Extraction and genotyping:

The QIAamp DNA Mini Blood Kit (Qiagen, Hilden, Germany) was used to extract genomic DNA from blood leukocytes according to the manufacturer’s instructions. Only samples with an A260/A280 ratio between 1.8 and 2.0 were included. DNA concentration and purity were determined using a spectrophotometer (UV-1900i Plus, SHIMADZU, Kyoto, Japan). Subsequently, the isolated DNA was stored at -20°C until further analysis.

### Primers:

For *PON1* Q192R (rs662)

- Forward: 5′-TATTGTTGCTGTGGGACCTGAG-3′
- Reverse: 5′-CACGCTAAACCCAAATACATCTC-3′

For *PON1* L55M (rs854560)

- Forward: 5′-GCTCTAGTCCATCAATTTAAAACAAA-3′
- Reverse: 5′-TGGGTATACAGAAAGCCTAAGTGA-3′

### Genotyping for PON1 polymorphisms:

Initial denaturation occurred at 94°C for four minutes, followed by 40 cycles of denaturation at 94°C for one minute, primer annealing at 56 °C for one minute, and extension at 72°C for one minute, concluding with a final five minute extension at 72°C. The PCR products for the L55M and Q192R polymorphisms were cleaved with (NlaIII) and (AlwI) restriction enzymes from New England BioLabs (Ipswich, MA, USA). For the L55M SNP, PCR-RFLP electrophoresis categorized genotypes as follows: the homozygous LL genotype (wild-type) produced an uncleaved 394 bp band; the homozygous MM genotype showed 124 bp and 245 bp fragments; and the heterozygous LM genotype displayed all three fragments (124 bp, 245 bp, and 394 bp). For Q192R genotyping, the heterozygous QR genotype was indicated by all three fragments (35 bp, 63 bp, and 98 bp); the homozygous RR genotype appeared with 35 bp and 63 bp fragments; and the homozygous QQ genotype (wild-type) was characterized by a single uncut 98 bp band. PCR-RFLP for the *PON1* Q192R polymorphism utilized 4% agarose gel electrophoresis.^11^

### Ethics approval:

Obtained from the “Research Ethics Committee” at Taibah University (Approval No. CLS 2020105; dated December 20, 2020). This committee follows the guidelines of the “1964 Helsinki Declaration and all its amendments. Before participating, all participants in this study signed an informed consent.

### Statistical analysis:

The analysis was conducted using SPSS version 23; continuous variables were reported as mean ± standard deviation (SD). Unpaired Student’s t-test, ANOVA, and Chi-square/Fisher exact test, were employed as appropriate. In addition, a *p*-value <0.05 was considered a statistically significant difference.

## RESULTS

The baseline characteristics of the study population, including demographic, clinical, and biochemical parameters, comprising 220 patients with T2DM and 120 healthy controls matched for age and sex. Age, sex distribution, smoking status, and blood pressure did not differ significantly between the groups (p > 0.05) are presented in Table-I.. In contrast, BMI, PON1, FPG, HbA1c, TC, TG, LDL, and apoB were significantly higher in cases than in controls (*p* < 0.001). In addition, HDL and apo A1 were lower in cases than in controls (*p* < 0.001).

**Table-I:** Basic data of study population.

Parameters	Cases (n=220)	Controls (n=120)	P-value
Gender (males, females)	135, 85	70, 50	0.914
Age (year)	56±10.16	54.1±9.1	0.088
BMI	25.2 ±3.3	23.1±1.29	<0.001*
***Blood pressure***			
SBP (mm/Hg)	120.9 ±12.15	119 ±10.2	0.14
DBP (mm/Hg)	81.1±6.9	80.3±4.1	0.24
Hypertensive patients	69 (31.4 %)	-	
Smoking	72 (32.7 %)	31 (25.8 %)	0.084
PON1 (ng/ml)	62.2±6.5	98.8±11.2	<0.001*
FPG (mmol/l)	7.2±1.2	5.04±0.61	<0.001*
HbA1c %	7.3±1.19	4.9±0.49	<0.001*
***Lipids***			
TC (mmol/l)	4.16±0.29	3.52±0.2	<0.001*
TG (mmol/l)	1.6±0.1	1.33±0.1	<0.001*
LDL (mmol/l)	2.59±0.2	2.29±0.1	<0.001*
HDL (mmol/l)	1.18±0. 1	1.25±0.05	<0.001*
Apo A1 (g/l)	1.2±0.1	1.65±0.1	<0.001*
Apo B (g/l)	12.9±3.5	9±1.2	<0.001*
Nephropathy	19 (8.6%)	-	-
Retinopathy	13 (5.9%)	-	-
Cardiovascular diseases	19 (8.6%)	-	-
Neuropathy	9 (4.1%)	-	-

* Significant when compared between cases and controls (p<0.001).

***Abbreviations:*** BMI: body mass index. NW: normal weight. OW: overweight. OB: obese. SBP: systolic blood pressure. DBP: diastolic blood pressure. PON1: Paraoxonase-1. FPG: fasting plasma glucose. HbA1c: hemoglobin A1c. TC: Total cholesterol. TG: triglyceride. LDL: low-density lipoprotein cholesterol. HDL: High-density lipoprotein cholesterol.

The percentage distribution of *PON1* Q192R and L55M genotypes and alleles among patients with T2DM and controls are presented in Table-II.. For Q192R, the QR genotype was most common in patients (43.6%) and controls (48.3%), followed by QQ (40.0% vs. 42.5%) and RR (16.4% vs. 9.2%), with no significant difference between the groups (*p* = 0.18). For L55M, the LL genotype was more common in patients (50.9%) and controls (53.3%), followed by LM (39.1% vs. 40.0%) and MM (10.0% vs. 6.7%), with no statistically significant difference (*p* = 0.58). Allele analysis showed that the Q allele (61.8%) and L allele (70.5%) were more common in patients than the R (38.2%) and M (29.5%) alleles, and these frequencies were similar to those in controls (*p* > 0.05). The genotype distributions in both groups were consistent with Hardy-Weinberg equilibrium.

**Table-II:** Distribution of PON1 192 and 55 gene polymorphisms, genotypes and alleles in patients with T2DM diabetic and controls.

Genotype	Cases (n=220)	Controls (n=120)	P-value	HWE
***PON1 192***				0.338
RR	36 (16.4%)	11 (9.2%)		
QR	96 (43.6%)	58 (48.3%)	0.18	
QQ	88 (40 %)	51 (42.5%)		
***PON1 55***				0.8
LL	112 (50.9%)	64 (53.3%)		
LM	86 (39.1%)	48 (40%)	0.58	
MM	22 (10%)	8 (6.7%)		
***Allele***				
R	168 (38.2%)	80 (33.3%)	0.21	
Q	272 (61.8%)	160 (66.7%)		
***Allele***				
L	310 (70.5%)	176 (73.3%)	0.43	
M	130 (29.5%)	64 (26.7%)		

HWE: Hardy-Weinberg equilibrium.

Correlations among clinical and biochemical markers in patients with T2DM and *PON1* Q192R and L55M genotypes is shown in Table-III. The Q192R polymorphism was strongly associated with the incidence of cardiovascular disease (*p* = 0.04) but not with nephropathy, retinopathy, neuropathy, smoking, or hypertension. Moreover, the Q192R and L55M polymorphisms were substantially associated with serum PON1 levels and glycemic indices (HbA1c and FPG); carriers of the QQ and MM genotypes had lower PON1 concentrations and poorer glycemic control (*p* < 0.05 - 0.01).

**Table-III:** Distribution of PON1 192 55 gene polymorphisms, genotypes and alleles in patients with T2DM with different clinical and biochemical variables.

Item	Status(n)	PON1 192 genotype				PON1 55 genotype			
		RR (36) n (%)	QR (96) n (%)	QQ (88) n (%)	P-value	LL (112) n (%)	LM (86) n (%)	MM (22) n (%)	P-value
Nephropathy	Yes (19)	2 (5.5)	11 (11.5)	6 (6.8)	0.41	9 (8)	6 (7)	4 (18.2)	0.23
	No (201)	34 (94.5)	85 (88.5)	82 (93.2)		103 (92)	80 (93)	18 (81.8)	
Retinopathy	Yes (13)	2 (5.5)	8 (8.53)	3 (3.4)	0.36	7 (6.3)	3 (3.5)	3 (13.6)	0.19
	No (207)	34 (94.5)	88 (91.7)	85 (96.6)		105 (93.7)	83 (96.5)	19 (86.4)	
Cardiovascular diseases	Yes (19)	6 (16.7)	10 (10.4)	3 (3.4)	0.04*	10 (8.9)	5 (5.8)	4 (18.2)	0.18
	No (201)	30 (83.3)	86 (89.6)	85 (96.6)		102 (91.1)	81 (94.2)	18 (81.8)	
Neuropathy	Yes (9)	1 (2.8)	6 (6.3)	2 (2.3)	0.36	5 (4.5)	2 (2.3)	2 (9.1)	0.35
	No (211)	35 (97.2)	90 (93.7)	86 (97.7)		107 (95.5)	84 (97.7)	20 (90.9)	
Smoking	Yes (72)	6 (16.7)	34 (35.4)	32 (36.4)	0.08	36 (32.1)	30 (34.9)	6 (27.3)	0.78
	No (148)	30 (83.3)	62 (64.6)	56 (63.6)		76 (67.9)	56 (65.1)	16 (72.7)	
Hypertension	Yes (69)	6 (16.7)	32 (33.3)	31 (35.2)	0.11	35 (31.3)	30 (34.9)	4 (18.2)	0.32
	No (151)	30 (83.3)	64 (66.7)	57 (64.8)		77 (68.7)	56 (65.1)	18 (81.8)	
		*mean±SD*				*mean±SD*			
BMI		25.3±3.9	25.1±2.0	24.3±1.82	0.07	25.2±2.1	25.3±3.5	24.6±1.5	0.55
TC		4.61±1.1	4.59±1	4.31±0.47	0.058	4.6±1.2	4.47±0.9	4.32±0.3	0.43
LDL		2.9±1.0	2.8±0.9	2.65±0.33	0.09	2.87±0.9	2.8±0.9	2.56± 0.33	0.30
HDL		1.06±0.2	1.1±0.1	1.12±0.2	0.09	1.07±0.6	1.12±0.2	1.19±0.1	0.46
TG		1.82±0.2	1.8±0.2	1.77±0.1	0.12	1.81±0.2	1.8±0.2	1.72±0.1	0.13
Apo A1		1.32±0.5	1.27±0.2	1.22±0.05	0.16	1.35±0.4	1.3±0.2	1.25±0.1	0.29
Apo B		13.1±1.9	12.86± 1.8	12.55±1.1	0.06	12.9±2	12.86± 1.81	12.6±1.1	0.78
HbA1c		7.59±1.1	7.35±0.6	7.21±0.9	0.027*	7.46±0.6	7.3±0.6	7.12±0.4	0.02*
FPG		8.43±1.1	8.22±1.2	8.05±0.9	0.017*	8.45±0.9	8.2±0.8	8.07±0.4	0.03*
PON1		59.1± 11.7	67.3± 13.1	62.2±10.2	0.002**	68.9±12.5	67.1±10.5	61.5±9.1	0.008**

* Significant when compared between cases and controls *:(p<0.05, **:p<0.01). n: number. SD: standard deviation.

## DISCUSSION

In the present study, we investigated *PON1* Q192R and L55M polymorphisms, circulating PON1 concentration, and their associations with biochemical parameters and diabetic complications in Saudi patients with T2DM. The key findings were that serum PON1 levels were markedly reduced in patients with T2DM, that neither Q192R nor L55M polymorphisms were associated with T2DM susceptibility, and that the Q192R variant was significantly associated with cardiovascular disease (CVD) among diabetic patients. Collectively, these findings suggest that altered PON1 status in T2DM is driven primarily by metabolic and oxidative stress-related factors, whereas genetic variation in *PON1* may modulate vascular risk rather than diabetes onset.

The observed reduction in circulating PON1 levels in T2DM is consistent with a substantial body of evidence linking diabetes to impaired antioxidant defenses. PON1 plays a critical role in protecting lipoproteins from oxidative modification by hydrolyzing lipid peroxides and oxidized phospholipids. Reduced PON1 availability in T2DM may therefore exacerbate lipid peroxidation, promote endothelial dysfunction, and accelerate atherogenesis. This mechanism is biologically plausible and aligns with previous reports showing lower PON1 activity or concentration in diabetic populations, as well as an inverse relationship with markers of oxidative stress and poor glycemic control.^12^

The biochemical profile of the studied T2DM cohort-marked by hyperglycemia, dyslipidemia, and suboptimal metabolic control-provides a plausible explanation for the observed reduction in PON1. Chronic hyperglycemia and elevated triglyceride-rich lipoproteins are known to increase oxidative stress and may downregulate hepatic PON1 expression or impair enzyme stability on HDL particles. These findings are particularly relevant in the Saudi context, where the prevalence of T2DM and associated metabolic disturbances has risen sharply in recent decades,^13^,^14^ underscoring the contribution of environmental and metabolic factors to antioxidant dysfunction in this population.

In contrast, no significant differences were observed in the genotype or allele frequencies of the *PON1* Q192R or L55M polymorphisms between patients and controls, suggesting that these variants are unlikely to confer major susceptibility to T2DM in this cohort. This finding aligns with several previous studies reporting null associations between *PON1* polymorphisms and diabetes risk, despite clear reductions in enzyme activity or concentration among affected individuals.^14^-^20^ However, discrepant findings have also been reported in other populations,^21^ likely reflecting ethnic heterogeneity, differences in study design, and variation in environmental exposures. Indeed, meta-analyses indicate that although the Q192R and L55M polymorphisms influence PON1 enzymatic activity, their effects on disease risk are inconsistent across ethnic groups, underscoring the importance of population-specific investigations.

Notably, although *PON1* polymorphisms were not associated with diabetes susceptibility, a significant association was observed between the Q192R polymorphism and cardiovascular disease among patients with T2DM. Individuals carrying the QR genotype exhibited a higher prevalence of CVD compared with QQ homozygotes, suggesting that this variant may modify vascular risk in the diabetic state. This finding is biologically plausible, as the Q192R substitution alters PON1 catalytic efficiency and substrate specificity, particularly in the detoxification of lipid peroxides and oxidized LDL and HDL particles,^6^ Experimental and clinical studies have demonstrated that the R allele is associated with reduced protection against LDL oxidation, leading to increased oxidative burden and enhanced atherogenic potential.^22^

Previous epidemiological studies support this interpretation, reporting higher risks of coronary artery disease and adverse cardiovascular outcomes among R allele carriers, especially in individuals with diabetes or dyslipidemia.^23^ The diabetic milieu may further amplify the detrimental effects of the Q192R variant by increasing oxidative stress and weakening compensatory antioxidant mechanisms, thereby accelerating vascular damage.^24^ Taken together, these findings suggest that *PON1* Q192R may act as a genetic modifier of cardiovascular risk in T2DM rather than as a determinant of diabetes susceptibility per se.

Reduced PON1 levels are a more reliable indicator of metabolic and vascular imbalance in T2DM than the *PON1* genotype alone. While the Q192R variant may indicate higher cardiovascular risk, caution is warranted due to limited cardiovascular events and not assessing PON1 enzymatic activity. The differences between PON1 isoforms Q and R relate to cardiovascular disease but not T2DM susceptibility. The R isoform offers lower antioxidant protection and catalytic efficiency, suggesting it contributes more to vascular issues rather than glucose metabolism or insulin resistance. Thus, PON1 Q192R mainly modulates cardiovascular risk instead of being a direct factor in diabetes susceptibility.

The study investigates the relationship between circulating PON1 levels and *PON1* polymorphisms in Saudi individuals with T2DM. It implies that rather than genetic differences, lower levels of circulating PON1 may more accurately reflect oxidative and metabolic abnormalities. Given its correlation with cardiovascular illness, the Q192R polymorphism may have an impact on vascular risk in individuals with diabetes. Instead of predicting T2DM susceptibility, the results suggest that PON1 could be a biomarker for evaluating cardiometabolic risk. Although additional multicenter research is required to validate these results and investigate PON1’s function in diabetes complications, the study’s strengths include a thorough investigation of genetic and metabolic variables in a well-defined cohort.

### Limitations

Among limitations, generalizability and causal inference may be limited by the study’s hospital-based methodology, modest sample size, inclusion of a single Saudi ethnic population, and *Arylesterase* and enzymatic activity, which were not evaluated in this work, are intimately linked to the functional impact of *PON1* polymorphisms.

## CONCLUSION

The study shows that circulating PON1 levels are significantly lower in Saudi patients with T2DM, indicating increased oxidative stress and impaired antioxidant defense. While *PON1* Q192R and L55M polymorphisms were not linked to diabetes susceptibility, the Q192R variant was associated with cardiovascular disease, acting as a genetic modifier of vascular risk. The findings stress that metabolic dysregulation affects *PON1* status more than genetic factors, and future research is needed to assess if *PON1* may have potential as a biomarker in cardiovascular risk evaluation for T2DM.

### Recommendations

Further large-scale studies are needed to assess the clinical relevance of *PON1* genotyping in risk stratification for T2DM patients.

### Authors’ Contribution:

**AMA:** Sample collection, lab work, and writing. Takes complete responsibility for the project’s accuracy, integrity, and final approval.

**HHA and KA:** Statistical analysis, Literature review, and writing manuscript.

**HMA and ZE:** Interpretation, Critical analysis, writing, and reviewing the draft.

All authors are participating in all stages of research preparation.
